# Transactions of the Forty-fifth Session of the American Institute of Homœopathy

**Published:** 1893-09

**Authors:** 


TRANSACTIONS OF THE FORTY-FIFTH SESSION OF AMERICAN INSTITUTE OF HOMŒOPATHY, forty-eighth anniversary, held at Washington, D. C., June 13th to 17th, 1892. Edited by Pemberton Dudley, M. D., General Secretary. Philadelphia: Sherman & Co., Printers, Seventh and Cherry Streets, 1892.

These transactions form a fine volume of nearly eleven hundred pages. They were issued nearly three months ago. It has been the intention of the editor of this journal to give them an extended review; it is not possible to do so, and the intention is abandoned, and, instead, only this announcement of their being published is made, and with the statement that copies may be had of the Secretary, Dr. Pemberton Dudley, 1405 North Sixteenth Street, Philadelphia, Pa.

